# Use of insect repellent as personal protection among women of childbearing age in an arbovirus endemic area in Northeastern Brazil

**DOI:** 10.1590/1980-549720240025

**Published:** 2024-05-13

**Authors:** Livia Karla Sales Dias, Carlos Sanhueza-Sanzana, Francisco Marto Leal Pinheiro, Adriano Ferreira Martins, Francisco Gustavo Silveira Correia, Italo Wesley Oliveira de Aguiar, Nayane Cavalcante Ferreira, Jeni Stolow, George Rutherford, Maria Gloria Teixeira, Roberto da Justa Pires, Rosa Livia Freitas de Almeida, Ivo Castelo Branco Coelho, Cristiane Cunha Frota, Carl Kendall, Ligia Regina Franco Sansigolo Kerr

**Affiliations:** I Universidade Federal do Ceará, School of Medicine, Department of Community Health – Fortaleza (CE), Brazil.; II Tulane University, Tulane School of Public Health and Tropical Medicine, Department of Social Behavior and Population Science – New Orleans (LA), USA.; III University of California, Department of Epidemiology and Biostatistics – San Francisco, California (CA), USA.; IV Universidade Federal da Bahia, Institute of Collective Health – Salvador (BA), Brazil.; V Universidade Federal do Ceará, School of Medicine, Department of Pathology and Legal Medicine – Fortaleza (CE), Brazil.

**Keywords:** Public health, Arboviruses, Zika virus, Women’s health, Insect repellents, Vulnerable populations, Saúde pública. Arbovírus, Zika vírus, Saúde da mulher, Repelentes de insetos, Populações vulneráveis

## Abstract

**Objective:**

To analyze the factors associated with the individual use of insect repellent by women of childbearing age living in area endemic for arboviruses in Fortaleza, Brazil.

**Methods:**

This is a cohort study carried out between 2018 and 2019 with women aged between 15 and 39 years in Fortaleza, state of Ceará, Brazil. A total of 1,173 women users of one of the four selected primary health care units participated in the study. The outcome was divided into: continued use, discontinued use, and nonuse of insect repellent. Crude and adjusted multinominal logistic regression analysis was carried out guided by a hierarchical model, with presentation of the respective odds ratio (OR) and 95% confidence intervals (95%CI). The independent variables include: socioeconomic and demographic data, environmental and sanitary characteristics, knowledge of the insect repellent, and behavioral and pregnancy-related aspects.

**Results:**

Only 28% of the participants reported using insect repellent during the two waves of the cohort. Women with higher education (OR=2.55; 95%CI 1.44–4.51); who are employed (OR=1.51; 95%CI 1.12–2.03); who received guidance from healthcare professionals (OR=1.74; 95%CI 1.28–2.36) and the media (OR=1.43; 95%CI 1.01–2.02); who intensified precautions against mosquitoes during the epidemic (OR=3.64; 95%CI 2.29–5.78); and who were pregnant between 2016 and 2019 (OR=2.80; 95%CI 1.83–4.30) had increased odds for continued use of insect repellent.

**Conclusion:**

The use of insect repellent among women of childbearing age was associated with a higher level of education, employment, guidance on insect repellent provided by healthcare professionals and the media, behavioral changes to protect against mosquitoes during the Zika virus epidemic, and pregnancy when occurring as of the beginning of the epidemic period.

## RESUMO

**Objetivo:**

Analisar os fatores associados ao uso de repelente individual por mulheres em idade fértil residentes em área endêmicas para arboviroses em Fortaleza, Brasil.

**Métodos:**

Coorte realizada entre 2018 e 2019, com mulheres entre 15 e 39 anos, moradoras em Fortaleza, Ceará, Brasil. Participaram 1.173 mulheres, usuárias de quatro unidades primárias de saúde do município. O desfecho foi categorizado em: uso continuado, uso descontinuado e não uso de repelente. Realizada análise de regressão logística multinominal norteada por modelo hierárquico, com apresentação dos respectivos *odds ratio* (OR) e intervalos de confiança de 95% (IC95%). As variáveis independentes incluem: dados socioeconômicos e demográficos, características ambientais e sanitárias, conhecimento sobre o repelente e aspectos comportamentais e relacionados à gravidez.

**Resultados:**

Somente 28% das participantes referiram o uso de repelente durante as duas ondas da coorte. Mulheres com escolaridade superior (OR=2,55; IC95% 1,44–4,51); com emprego (OR=1,51; IC95% 1,12–2,03); que receberam orientações por profissionais da saúde (OR=1,74; IC95% 1,28–2,36) e pela mídia (OR=1,43; IC95% 1,01–2,02); que intensificaram os cuidados contra o mosquito na epidemia (OR=3,64; IC95% 2,29–5,78); estavam grávidas entre 2016 e 2019 (OR=2,80; IC95% 1,83–4,30) tiveram as chances aumentadas para uso continuado de repelente.

**Conclusões:**

O uso de repelente entre mulheres em idade fértil mostrou-se associado a um maior nível de escolaridade, ao emprego, às orientações sobre repelente fornecidas por profissionais de saúde e pela mídia, às mudanças comportamentais de cuidado contra o mosquito durante a epidemia da Zika e à gravidez quando ocorrida a partir do início do período epidêmico.

## INTRODUCTION

Arboviruses, including Dengue, Chikungunya, and Zika viruses, are major current threats to public health in tropical and subtropical areas, affecting about four billion people^1^. The global increase in the frequency and magnitude of arbovirus outbreaks has been driven by the convergence of ecological factors, including climate change^2,3^, and socioeconomic factors^4^.

These arboviruses have caused high morbidity and mortality, with a special impact in the Americas^5^. Brazil is one of the most affected countries with records of several Dengue epidemics in its history and, in recent years, it has been the center for Chikungunya and Zika^6,7^.

Socioeconomically vulnerable populations are more prone to mosquito-borne diseases due to precarious housing and basic sanitation infrastructure, failures in garbage collection, low access to information and healthcare services, low level of education, among other factors that hinder the practice of healthy actions and personal care, contributing to the permanence of these diseases^8,9^. The Zika virus (ZIKV) epidemic in 2015, both nationally and internationally^10,11^, highlighted these socioeconomic-environmental determinants when it mainly affected low-income, lower-educated, black women residents of peripheral regions of the Brazilian Northeast^9,12^.

The possibility of sexual and vertical transmission of ZIKV increased the risk of the disease and, as a result, personal and household care guidelines were intensified and aimed mainly at pregnant and/or childbearing women, who were the focus of attention during the epidemic^13,14^. In view of the difficulty in implementing vector control actions in the face of various socioeconomic and environmental issues and vaccine protection against arboviruses, the use of personal protection measures has become the most tangible action for the population to protect itself from diseases^15,16^.

The regular use of body repellent was one of the recommendations present in national and international handbooks, being directed mainly to pregnant women or those who intended to become pregnant, and especially if they lived in areas infested by *Aedes aegypti* ^17,18^.

Due to the epidemic scenario, in April 2016, the Brazilian government instituted the monthly distribution of two bottles of topical insect repellents to pregnant women in situations of socioeconomic vulnerability assisted by healthcare units^19^. In 2018, there was an expansion of the offer to all pregnant women and the population living in endemic areas^20^. However, with the reduction in cases of microcephaly and ZIKV infections in the country, in July 2019, the action was revoked by the government in force^21^.

In view of the recommendation of the use of insect repellent as a measure of personal protection against arboviruses, which was distributed to pregnant women, and considering the absence of studies on the panorama of the use of this measure when offered to pregnant women in the state of Ceará, which concentrates high numbers of cases of Dengue, Zika, and Chikungunya, in this study we aimed to analyze the factors associated with the individual use of insect repellent by women of childbearing age living in endemic areas for arboviruses in Fortaleza, Brazil.

## METHODS

### Study type and location

**Figure 1:**
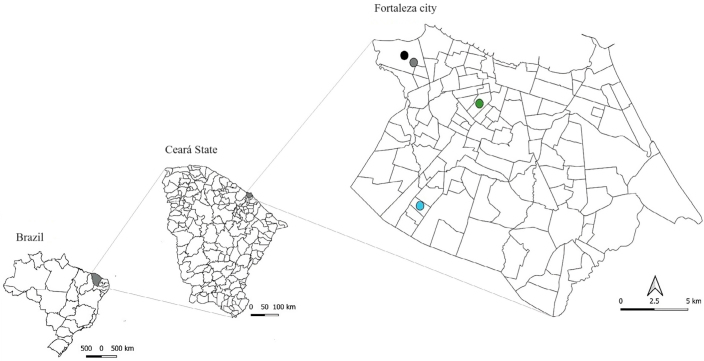
Location of the study area and selected health care units. Fortaleza (CE), Brazil, 2017.

This cohort study used data from the larger project entitled *Zika em Fortaleza: resposta de uma coorte de mulheres de 15 a 39 anos* (“Zika in Fortaleza: response of a cohort of women aged 15 to 39 years”) (ZIF project), carried out in the city of Fortaleza, state of Ceará, Brazil. In 2018, Fortaleza had an estimated population of 2,627,482 people with a population density of 8,373 inhabitants/km^2^, being divided into six administrative units (*Secretarias Executivas Regionais* [Regional Executive Secretariats] – SER). During the study period, there were 96 Primary Health Care Units (PHCUs), and four units located in SER I, III, and V were selected (Figure 1). These regions recorded high rates of Chikungunya cases in 2017, an infection taken as a proxy for ZIKV infection^22^.

### Sample calculation

Sexually active women aged between 15 and 39 years living in Fortaleza, state of Ceará, Brazil, participated in the study. The inclusion criteria were: 1) live in the territorial area of one of the selected health care units; 2) be between 15 and 39 years of age; 3) be sexually active (at least one sexual intercourse in the last 12 months). Women aged 15 to 39 years who had undergone tubal ligation and who lived outside the selected areas were excluded from the sample.

The sample size was estimated based on the equation:


$$\text{n}=[deff*\text{N}*(1-\text{P})]/[({\text{d}}^{2}/{\text{Z}}^{2}1-\alpha /2*(\text{n}-1)+\text{P}*(1-\text{P})]$$

The probability of pregnancy (P) was estimated at 8.3% (+/- 2%), dividing the total number of live births in the city by the sexually active female population, aged 15 to 39 years (N), estimated at 73% of the female population in the city of Fortaleza. Considering the design effect (*deff*) of 2, the 95% confidence interval and a loss to follow-up (d) of 20%, it resulted in a sample size (n) of 1,752 women.

Toward the end of the collection period, there was a reduction in the recruitment rate, considering that the pregnancy rate in the study population was higher (20%) than estimated (8.3%); therefore, the baseline sample was completed with 1,496 women.

### Data collection

Data collection took place in two waves. The meetings took place inside the health care units, and semi-structured questionnaires with similar questions were applied to the two waves, using the Survey Monkey software (SurveyMonkey Inc, San Mateo, California, USA). The collection period for the first wave was from February 28 to October 30, 2018; and the second wave, from February 14 to August 30, 2019.

### Dependent variable

The use of insect repellent during the cohort was considered as the variable of interest, and the following questions arose: “Do you use any kind of insect repellent?”; “Is the insect repellent for individual use?”, both with yes/no answers.

Subsequently, it was categorized into three levels: “Continued use,” when the use of insect repellent was reported in both waves of the cohort; “Discontinued use,” when the use was reported in only one of the cohort waves; and “Nonuse,” when the repellent was not used in both waves of the cohort.

### Independent variables

They were composed of:

- Socioeconomic and demographic factors:
  - Age group (years): variable initially continuous and later categorized into age groups: 15–19 (reference); 20–29; 30–39.
  - Race/Skin color: presented in five categories and later categorized into “white” and “non-white” (Black, mixed-race, Indigenous, Asian) (reference).
  - Level of education: based on the informed level of education, it was categorized into: Elementary school or lower (reference); Incomplete high school/Complete High school; Incomplete undergraduate/Complete undergraduate.
  - Employment status: categorized into “employed” and “unemployed” (reference).
- Environmental and sanitary characteristics of the household:

The variable presence of a backyard in the household was categorized as “yes” and “no” (reference). The variable household sewage destination was composed of: “public system/septic tank,” which are characterized as recommended sewage destinations, and “open air” (reference category).

- Received guidance on insect repellent:

The variables were based on the following questions: “Where did you receive guidance on the use of insect repellent as a protective measure?” Several means of information were presented to the participants, among them: healthcare professionals (physicians and non-physicians) and the media (the Internet and television), which were dichotomized into “yes” and “no” (reference).

- Zika-related behavioral factors:
  - Has postponed pregnancy due to the Zika epidemic: dichotomized into “ye” and “no” (reference);
  - Precaution against the disease-transmitting mosquito during the Zika epidemic: categorized as “intensified precautions against the mosquito” or “there were no behavioral changes” (reference).
- Pregnancy:

History of pregnancy before and as of the epidemic period was categorized as: pregnancy before 2016; pregnancy between 2016 and 2019; and has never gotten pregnant (reference). Childbirths/pregnancies that occurred before and from January 2016 onward were considered. This temporal demarcation was chosen because it was only after the Brazilian government declared an association between the Zika virus infection and cases of microcephaly, at the end of 2015^13,23^, that the use of personal protection measures for pregnant women and women of childbearing age began to be aimed at the prevention of the disease^24^.

### Statistical analysis

A descriptive analysis of the epidemiological profile of the participants and the characteristics of insect repellent use was performed, with presentation of the respective frequencies and confidence intervals (95%CI).

Nominal multinomial logistic regression was used to verify the associations of the independent variables with the response variable of interest, i.e., “Continued use,” “Discontinued use,” and “Nonuse” of insect repellent, the latter deemed as a reference category. The results were presented as odds ratios (OR) along with their respective 95% confidence intervals, considering a 5% significance level.

**Figure 2:**
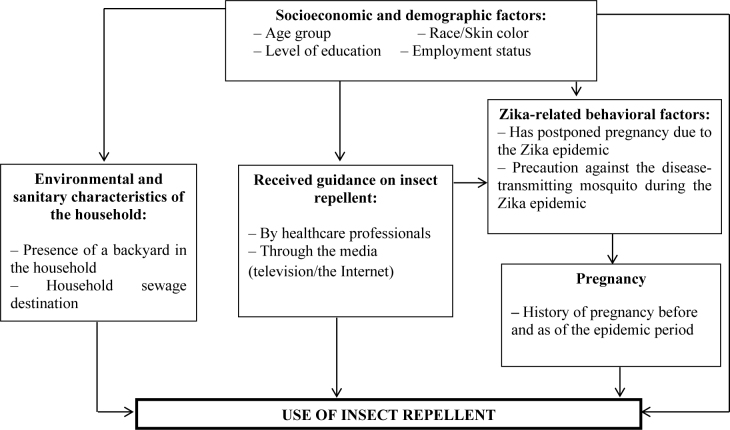
Hierarchical explanatory model for insect repellent use.

A hierarchical explanatory model was initially suggested for studies applied in other areas^25^. The predictor variables were grouped into five blocks, as shown in Figure 2: Block 1 – Socioeconomic and demographic factors (age group, level of education, race/skin color, employment status); Block 2 – Environmental and sanitary characteristics of the household (presence of a backyard in the household, household sewage destination); Block 3 – Received guidance on insect repellent (by healthcare professionals, through the media [television/the Internet]); Block 4 – Zika-related behavioral factors (has postponed pregnancy due to the Zika epidemic, precaution against the disease-transmitting mosquito during the Zika epidemic); Block 5 – Pregnancy (history of pregnancy before and as of the epidemic period).

Bivariate analyses were performed for all variables of the five blocks using Pearson’s chi-square hypothesis test (χ^2^). The socioeconomic and demographic variables (Block 1) were considered the most distal in the process of determining the use of the insect repellent. The variables of this block that were statistically significant at the 0.05 level remained in the final model. For the remaining blocks, the variables that remained significant up to 0.20 were evaluated in multinomial multivariate analysis internal to the block. The same process was repeated for the others. In the final model, all statistically significant variables were considered associated with the different uses of the insect repellent.

The Wald test was applied to evaluate the statistical relationship between the independent variable in the differentiation between the two groups and the likelihood ratio test to evaluate the overall relationship between the independent variables and the outcome. Statistical differences were compared between cohort waves for each exploratory variable using the Fisher’s exact test. The variables age group, ethnicity/skin color, and level of education were analyzed based on information provided in the 1st wave, and the variables in block 4 were investigated only in the 1st wave, being analyzed based on this information (Supplementary Table 1). All analyses were performed using the STATA™ software v. 16.

### Ethical aspects

This investigation is part of a matrix project approved by the Research Ethics Committee of the Universidade Federal do Ceará (UFC), under protocol No. 2.497.069, complying with national and international ethical guidelines for research involving human beings.

## RESULTS

A total of 1,496 women of childbearing age participated in the first wave, in 2018. In the second wave (2019), there was a loss to follow-up of 323 women (21.6%), totaling 1,173 participants, who were analyzed in this article.

Regarding the epidemiological profile of the participants (Table 1), most were between 20 and 29 years old (48.6%), self-reported to be mixed-race (70.3%), had incomplete high school or high school education (57.7%), and had a steady partner (60.4%). Regarding employment status, 61% were unemployed. About 78% of the women had a history of pregnancy, with the majority of pregnancies occurring between 2016 and 2019.

**Table 1:** Epidemiological profile and insect repellent use of a cohort of women of childbearing age. Fortaleza (CE), Brazil. 2018–2019.

Variable	n (%)	CI (95%)
Age group (years)		
15–19	240 (20.5)	18.2–22.9
20–29	570 (48.6)	45.7–51.5
30–39	363 (30.9)	28.4–33.6
Level of education		
Illiterate/Incomplete elementary school	291 (24.8)	22.4–27.4
Elementary school	103 (8.8)	7.3–10.5
Incomplete High school/Complete High school	676 (57.7)	54.8–60.5
Incomplete undergraduate /Complete undergraduate	102 (8.7)	7.2–10.5
Race/Skin color		
Mixed-race	830 (71.1)	68.4–73.6
White	126 (10.8)	9.1–12.7
Black	119 (10.2)	8.6–12.1
Others (Indigenous/Asian)	92 (7.95)	6.5–9.6
Employment status		
Employed	457 (39.0)	36.2–42.0
Unemployed	716 (61.0)	59.2–64.0
Marital status		
Married/Living with a partner	708 (60.4)	36.9–42.5
Single/Without a steady partner	465 (39.6)	57.5–63.1
History and periods of pregnancy		
Pregnancy before 2016	351 (29.9)	27.4–32.6
Pregnancy between 2016 and 2019	562 (47.9)	45.0–51.0
Has never gotten pregnant	260 (22.2)	20.0–24.6
Use of insect repellent in the cohort		
Only in the 1st wave	191 (16.3)	14.3–18.5
Only in the 2nd wave	205 (17.4)	15.4–19.8
Use in the 1st and 2nd waves	327 (28.0)	25.4–30.5
Nonuse	449 (38.3)	35.6–41.1

95%CI: 95% confidence interval.

Regarding the use of insect repellent during the cohort, approximately 62% of the women followed the recommendation at some point during the follow-up, of which 28% reported the use of insect repellent in both waves, while 38.3% did not use it.

The main justifications for not using repellent were: “do not believe it is necessary” (37.2%); “very expensive” (22.0%); “do not like to use it” (11.5%), the latter being associated with the consistency and smell of the product. Other factors, such as “forgot to use it” (9.4%), “sloppiness/carelessness” (5.3%), were also reported. In addition, 1.6% of the women denied the current use, but reported having used it at specific times: “at times when more mosquitoes appear,” during “epidemics.” Another reported factor was “fear of using the insect repellent,” which was related to the fact that they were pregnant or because they had some underlying disease. Other justifications were related to the replacement of the insect repellent by other products, such as creams, moisturizers and sunscreen, and by mechanical methods such as fans and mosquito nets.

In Table 2 we show the bivariate analysis between the independent variables and the outcome categories. From the analysis of the blocks, the variables level of education and employment status of block 1 remained statistically significant. The variables in block 2 remained significant only for the category of discontinued use. In block 3, guidance provided by healthcare professionals and the media was significant when jointly analyzed. In block 4, only the variable precaution against the disease-transmitting mosquito during the Zika epidemic was significant for the outcome categories. Finally, in block 5, the variable history of pregnancy before and as of the epidemic period was extremely significant for the continued and discontinued use of the insect repellent.

**Table 2:** Bivariate analysis between the independent variables and the categories of continued use, discontinued use, and nonuse of repellent. Fortaleza (CE), Brazil. 2018–2019.

Variable	Continued use	Discontinued use	Nonuse	p-value
	n (%)	n (%)	n (%)	
**Block 1 – Socioeconomic and demographic factors**				
Age group (years)				
15–19	59 (24.6)	87 (36.2)	94 (39.2)	0.406
20–29	169 (29.7)	194 (34.1)	206 (36.2)	
30–39	99 (27.3)	115 (31.7)	149 (41.0)	
Race/Skin color				
Nonwhite	283 (27.2)	357 (34.3)	400 (38.5)	0.306
White	42 (33.3)	37 (29.4)	47 (37.3)	
Level of education				
Elementary school or lower	91 (23.2)	135 (34.3)	167 (42.5)	0.005
Incomplete High school/Complete High school	196 (29.0)	223 (33.0)	257 (38.0)	
Incomplete undergraduate /Complete undergraduate	39 (38.2)	38 (37.3)	25 (24.5)	
Employment status				
Employed	148 (32.4)	157 (34.3)	152 (33.3)	0.005
Unemployed	179 (25.0)	239 (33.4)	297 (41.6)	
**Block 2 – Environmental and sanitary characteristics of the household**				
Presence of a backyard in the household				
Yes	174 (27.2)	232 (36.4)	232 (36.4)	0.115
No	153 (28.7)	164 (30.7)	217 (40.6)	
Household sewage destination				
Public system/septic tank	299 (27.8)	371 (34.6)	404 (37.6)	0.149
Open air	28 (28.6)	25 (25.5)	45 (45.9)	
**Block 3 – Received guidance on insect repellent**				
By healthcare professionals				
Yes	207 (33.0)	210 (33.4)	211 (33.6)	≤0.001
No	120 (22.1)	186 (34.2)	238 (43.7)	
Through the media (television/the Internet)				
Yes	253 (31.0)	271 (33.2)	292 (35.8)	0.001
No	74 (20.8)	125 (35.1)	157 (44.1)	
**Block 4 – Zika-related behavioral factors**				
Has postponed pregnancy due to the Zika epidemic				
Yes	38 (33.0)	39 (34.0)	38 (33.0)	0.307
No	281 (27.0)	355 (34.0)	407 (39.0)	
Precaution against the disease-transmitting mosquito during the Zika epidemic				
Intensified mosquito precautions	298 (30.8)	335 (34.6)	335 (34.6)	≤0.001
There were no behavioral changes	27 (13.6)	59 (29.8)	112 (56.6)	
**Block 5 – Pregnancy**				
History of pregnancy before and as of the epidemic period				
Pregnancy before 2016	87 (24.9)	97 (27.7)	166 (47.4)	≤0.001
Pregnancy between 2016 and 2019	187 (33.3)	212 (37.7)	163 (29.0)	
Has never gotten pregnant	53 (20.4)	87 (33.5)	120 (46.1)	

The final model of the multinomial logistic regression is presented in Table 3. Women of childbearing age with a college degree were more likely to continue using the insect repellent (OR=2.55; 95%CI 1.44–4.51) and to discontinue its use (OR=1.77; 95%CI 1.01–3.10), in relation to those with elementary level of education or lower. Being employed had a positive impact on the use of the measure throughout the follow-up (OR=1.51; 95%CI 1.12–2.03) compared to unemployed women. Receiving guidance on insect repellent from healthcare professionals (OR=1.74; 95%CI 1.28–2.36) and the media (OR=1.43; 95%CI 1.01–2.02) had repercussions on the chances of continued use of the measure. Women who intensified their precautions against the mosquito during the epidemic had increased chances of both continued use of the insect repellent (OR=3.64; 95%CI 2.29–5.78) and the discontinued use (OR=1.86; 95%CI 1.30–2.65), compared to participants who did not change their behaviors. Pregnant women between 2016 and 2019 were more likely to continue using the repellent (OR=2.80; 95%CI 1.83–4.30) and to discontinue using it (OR=1.88; 95%CI 1.30–2.72) in relation to women who had never gotten pregnant.

**Table 3:** Multinomial logistic regression of factors associated with insect repellent use in a cohort of women aged 15 to 39 years. Fortaleza (CE), Brazil. 2018–2019.

Variables	Block 1	Block 2	Block 3	Block 4	Block 5
	Socioeconomic and demographic factors	Environmental and sanitary characteristics of the household	Received guidance on insect repellent	Zika-related behavioral factors	Pregnancy
Continued use	OR (95%)	OR (95%)	OR (95%)	OR (95%)	OR (95%)
Elementary school or lower	1				
Incomplete High school/Complete High school	1.37 (0.99–1.88)				
Incomplete undergraduate / Complete undergraduate	2.55 (1.44–4.51)*				
Employed	1.51 (1.12–2.03)^†^				
Presence of a backyard in the household		1.05 (0.78–1.41)			
Sewage destination by public system/septic tank		0.93 (0.51–1.57)			
Received guidance on insect repellent by healthcare professionals			1.74 (1.28–2.36)*		
Received guidance on insect repellent through the media			1.43 (1.01–2.02)^‡^		
Intensified mosquito precautions during the Zika epidemic				3.64 (2.29–5.78)*	
Pregnancy before 2016					1.11 (0.71–1.75)
Pregnancy between 2016 and 2019					2.80 (1.83–4.30)*
Has never gotten pregnant					1
**Discontinued use**	**OR (95%)**	**OR (95%)**	**OR (95%)**	**OR (95%)**	**OR (95%)**
Incomplete High school/ Complete High school	1.06 (0.79–1.42)				
Incomplete undergraduate / Complete undergraduate	1.77 (1.01–3.10)^‡^				
Employed	1.23 (0.93–1.63)				
Presence of a backyard in the household		1.30 (0.98–1.72)			
Sewage destination by public system/septic tank		1.62 (0.96–2.73)			
Received guidance on insect repellent by healthcare professionals			1.23 (0.92–1.63)		
Received guidance on insect repellent through the media			1.01 (0.75–1.37)		
Intensified mosquito precautions during the Zika epidemic				1.86 (1.30–2.65)*	
Pregnancy before 2016					0.77 (0.51–1.14)
Pregnancy between 2016 and 2019					1.88 (1.30–2.72)^‡^
Has never gotten pregnant					1

Multinomial Logistic Regression reference category: “Nonuse of insect repellent”; OR: odds ratio; 95%CI: 95% confidence interval. *p≤0.001; ^†^p≤0.01; ^‡^p≤0.05.

## DISCUSSION

Studies carried out during the ZIKV epidemic documented the relationship between the level of education in the face of behavioral changes and the population’s knowledge of the prevention of arboviruses. Authors of these studies identified that a lower level of education led to low use of recommended precautions^26,27^, while a higher level of education and better economic conditions favored the adoption of personal protection measures^28-30^, corroborating this research. However, approximately 9% of the participants in this survey reported having a college degree, a number far below the Brazilian level — according to which, in 2019, there were 19.4% women aged 25 years and over who had a college degree^31^, demonstrating that the study participants compose a group of high social vulnerability.

In Brazil, the targeting of public policies to pregnant women during the Zika epidemic, including the delivery of insect repellent, guidance in prenatal guides, and the dissemination of information through the media^13,23^, made pregnancy a prominent factor in the association with insect repellent use, as evidenced in this study. Countries affected by the ZIKV epidemic also highlighted this finding. Authors of a research conducted in Puerto Rico during the epidemic period found that pregnant women were 1.44 times more likely to use insect repellent than nonpregnant women^32^. In the Virgin Islands, there was a high rate of adherence to insect repellent by pregnant women (74%), who received educational materials and home and personal protection resources, including insect repellent^33^.

In this cohort, despite the strong association between pregnancy and insect repellent use, we observed a low percentage of continued repellent use by pregnant women between 2016 and 2019, compared to that found in the Virgin Islands. It is also noteworthy that 29% of these pregnant women did not adhere to the measure despite receiving the product free of charge during the period, suggesting flaws in the guidance on the supply and use of the insect repellent, in addition to other justifications for nonuse found in this study.

The participants of the research, pregnant and nonpregnant, evidenced as the main justifications for nonadherence the insufficient knowledge of the repellent and the financial aspect linked to its acquisition. During the ZIKV epidemic, the prevention of the disease entailed costs for women, leading the poorest to put aside practices that required financial expenditure^30,34^. This is the case of insect repellents, which, when not offered free of charge, implies additional costs for the family.

According to Dorsett et al.^35^, the voluntary use of insect repellent would not lead to the eradication of the disease; however, when its access is enabled and expanded, either at more affordable costs or distributed free of charge, its effectiveness increases and provides greater protection to the community.

Taking this into consideration, the discontinuity of the insect repellent supply policy in Brazil in 2019 will impact access to the measure, especially by the most disadvantaged women, increasing the risk of exposure to the virus and the possibility of new epidemics. The end of the insect repellent distribution policy occurred one month before the end of the cohort’s data collection, making it impossible to analyze the impact on access to the measure after this period.

Accessing the product does not warrant its use. In this study, guidance provided by healthcare professionals and the media favored the use of insect repellent, a fact also evidenced in other studies^28,30^. Conversely, the information only seemed to make a difference for a portion of the women exposed to the recommendations. Authors of a study conducted in Colombia between 2017 and 2018 identified a high level of knowledge of Zika and its forms of transmission among pregnant women; nonetheless, these factors did not result in an impact on changing behavior and adherence to protective measures^36^.

The provision of guidance without considering the characteristics of the target audience, behavioral changes, and the application of advances in the theory and practice of health communication^27,34^ is reflected in low adherence to the measure or irregular use, even when a preventive strategy is accessed^28,29,37^.

It should be noted that after the drop in Zika cases and births of children with congenital syndromes related to the Zika virus, there was a reduction in the spread of guidelines to combat arboviruses. Nevertheless, the coping with the Sars-Cov-2 pandemic situation in 2020 camouflaged the attention to arboviruses^38^, causing an underreporting of cases and relaxation of public health measures.

Given this scenario, Brazil has been registering an increase in cases of Dengue, Chikungunya, and Zika^39,40^. The increase in cases linked to the socioeconomic conditions of the population aggravated by the COVID-19 pandemic could turn Zika and other arboviruses into a true social, economic, and public health disaster in the country, if they are not prevented and controlled.

This study has limitations, among them the homogeneity of the sample, which, due to the purposes of the study, does not enable to extrapolate the data to groups with greater socio-environmental and economic advantage. Furthermore, there may be biases of loss to follow-up that occurred in the second wave, even after attempts to contact them through telephone calls and support from health agents. We identified statistical differences between the waves for the variables employment status and guidance provided by healthcare professionals and the media. In addition to the losses to follow-up, we understand that Brazil’s economic instability was reflected in the high unemployment rates, which were aggravated during the cohort period. Regarding the guidelines for the use of insect repellent, there was a decrease in the dissemination of information as it was distancing itself at the beginning of the epidemic, causing differences between the waves of the cohort. Moreover, the collected pieces of information were self-reported by the participants, and there may be memory biases; in order to minimize them, we used recall methods during the interview, and the participants were reminded of the years related to the epidemic and related events. Finally, the identification of the use of insect repellent did not consider the frequency of applications, and it was not possible to guarantee that it occurred according to the product’s recommendations^41^.

Despite these limitations, this study has the potential to support further research by presenting relevant findings and guiding government agencies in the formulation of public policies for the prevention of arboviruses. By adopting the cohort design, we identified factors that influence, over time, the use of insect repellent as a measure of personal protection among women of childbearing age, pregnant and nonpregnant.

All in all, we conclude this study by stating that the use of insect repellent as a measure of personal protection among women of childbearing age was associated with a higher level of education, employment, guidance provided by healthcare professionals and the media, and especially pregnancy and behavioral changes aimed at precaution against Zika and other arboviruses. However, the socioeconomic profile linked to the use of the measure does not represent a large portion of the Brazilian population, making it necessary to strengthen public policies that guarantee access to education, services and recommended means of personal and home protection. Taking this into consideration, we recommend the provision of insect repellent to women of childbearing age, living in endemic areas and with low socioeconomic status, combined with the provision of safe and reliable guidance, using an accessible language and which is feasible to the reality of these women and families, in order to strengthen knowledge and adherence to the measure and mitigate the high risk of exposure to the virus.
