# Supplementary material for: Use of insect repellent as personal protection among women of childbearing age in an arbovirus endemic area in Northeastern Brazil
Source: Rev Bras Epidemiol. 2024 May 13;27:e240025. doi: 10.1590/1980-549720240025 (PMC11093518; doi:10.1590/1980-549720240025)
Supplement: Supplementary file 1 [file 1980-5497-rbepid-27-e240025-Material-suplementar.docx]

**Tabela Suplementar 1. Distribuição das variáveis independentes entre as ondas de uma coorte de mulheres de 15 a 39 anos.**

**Fortaleza, Ceará, Brasil. 2018-2019.**

| **Variável** | **1ª Onda (N=1.496)** | | **2ª Onda (N=1.173)** | |  |
| --- | --- | --- | --- | --- | --- |
|  | **n (%)** | **IC(95%)** | **n (%)** | **IC(95%)** | ***p-valor* ^e^** |
| **Bloco 1 - Fatores socioeconômicos e demográficos** |  |  |  |  |  |
| Faixa etária (anos) |  |  |  |  | 0,521 |
| 15-19 | 311 (20,8%) | 18,8 – 22,9 | 233 (19,9%) | 17,7 – 22,2 |  |
| 20-29 | 742 (49,6%) | 47,1 – 52,1 | 569 (48,5%) | 45,6 – 51,4 |  |
| 30-39 | 443 (29,6%) | 27,3 – 32,0 | 371 (31,6%) | 29,0 – 34,3 |  |
| Raça/Cor |  |  |  |  | 0,664 |
| Não branca | 1.318 (66,6%) | 86,9 – 90,1 | 126 (10,8%) | 87,3 – 91,0 |  |
| Branca | 169 (11,4%) | 9,8 – 13,1 | 1.041 (89,2%) | 9,1 – 12,7 |  |
| Escolaridade |  |  |  |  | 0,763 |
| Elementar completo ou menos | 513 (34,3%) | 32,0 – 36,8 | 394 (33,6%) | 31,0 – 36,4 |  |
| Médio incompleto/completo | 843 (56,4%) | 54,0 – 59,0 | 676 (57,7%) | 54,8 – 60,5 |  |
| Superior incompleto/completo | 139 (9,3%) | 7,9 – 11,0 | 102 (8,7%) | 7,2 – 10,5 |  |
| Situação de trabalho |  |  |  |  | ≤0,001 |
| Com emprego | 526 (35,2%) | 32,8 – 37,6 | 277 (23,6%) | 21,3 – 26,1 |  |
| Sem emprego | 970 (64,8%) | 62,4 – 67,2 | 896 (76,4%) | 73,9 – 78,7 |  |
| **Bloco 2 - Características ambientais e sanitárias do domicílio** |  |  |  |  |  |
| Presença de quintal no domicílio |  |  |  |  | 1,000 |
| Sim | 766 (51,3%) | 48,8 – 53,9 | 602 (51,4%) | 48,5 – 54,2 |  |
| Não | 726 (48,7%) | 46,1 – 51,2 | 570 (48,6%) | 45,8 – 51,5 |  |
| Destino do esgoto do domicílio |  |  |  |  | 0,726 |
| Sistema público/Fossa séptica | 1.344 (94,4%) | 93,1 – 95,5 | 1.062 (94,7%) | 93,3 – 95,9 |  |
| Céu aberto | 80 (5,6%) | 4,5 – 6,9 | 59 (5,3%) | 4,1 – 6,7 |  |
| **Bloco 3 - Recebeu orientação sobre repelente** |  |  |  |  |  |
| Por profissionais da saúde |  |  |  |  | ≤0,001 |
| Sim | 767 (51,3%) | 48,7 – 53,8 | 27 (2,3%) | 1,6 – 3,3 |  |
| Não | 724 (48,7%) | 46,2 – 51,3 | 1.146 (97,7%) | 96,7 – 98,4 |  |
| Através da mídia (televisão/internet) |  |  |  |  | ≤0,001 |
| Sim | 1.025 (68,5%) | 66,1 – 70,8 | 59 (5,0%) | 3,9 – 6,4 |  |
| Não | 471 (31,5%) | 29,2 – 33,9 | 1.114 (95,0%) | 93,6 – 96,1 |  |
| **Bloco 4 - Fatores comportamentais relacionados à Zika** |  |  |  |  |  |
| Adiou a gestação devido à epidemia da Zika |  |  |  |  | 0,895 |
| Sim | 144 (9,7%) | 8,3 – 11,4 | 115 (9,9%) | 8,3 – 11,8 |  |
| Não | 1.333 (90,3%) | 88,6 – 91,7 | 1.044 (90,1%) | 88,2 – 91,7 |  |
| Cuidados contra o mosquito transmissor durante a epidemia da Zika |  |  |  |  | 0,571 |
| Intensificou os cuidados contra  o mosquito | 1.221 (82,2%) | 80,1 – 84,0 | 969 (83,0%) | 81,0 – 85,1 |  |
| Não houve mudanças  comportamentais | 265 (17,8%) | 16,0 – 20,0 | 198 (17,0%) | 14,9 – 19,2 |  |
| **Bloco 5 - Gravidez** |  |  |  |  |  |
| História de gravidez antes e partir do período epidêmico |  |  |  |  | 0,982 |
| Gravidez antes de 2016 | 442 (29,6%) | 27,3 – 32,0 | 351 (29,9%) | 27,4 – 32,6 |  |
| Gravidez entre 2016 e 2019 | 720 (48,2%) | 45,7 – 51,0 | 562 (47,9%) | 45,1 – 51,0 |  |
| Nunca engravidou | 331 (22,2%) | 20,1 – 24,3 | 260 (22,2%) | 20,0 – 24,6 |  |

IC95%: Intervalo de Confiança de 95%

^e^Diferenças estatísticas entre as ondas da coorte em relação a linha de base e após as perdas de seguimento utilizando teste exato de Fisher.
